# Potential Distribution of Tribe Erythroneurini in China Based on the R-Optimized MaxEnt Model, with Implications for Management

**DOI:** 10.3390/insects16050450

**Published:** 2025-04-24

**Authors:** Xiaojuan Yuan, Weiwei Ran, Wenming Xu, Yuanqi Zhao, Di Su, Yuehua Song

**Affiliations:** 1 School of Karst Science, Guizhou Normal University, Guiyang 550025, China; yxj00816@163.com (X.Y.); ww_ran@163.com (W.R.); 232100170559@gznu.edu.cn (W.X.); zyq19991208@163.com (Y.Z.);; 2 State Engineering Technology Institute for Karst Desertification Control, Guiyang 550025, China

**Keywords:** climate change, geographical distribution pattern, pest management, species distribution modeling, Typhlocybinae

## Abstract

This study examines the distribution dynamics of the tribe Erythroneurini, a group of economically significant leafhoppers in China that pose threats to crops through sap feeding and virus transmission, while also serving as valuable ecological indicators due to their sensitivity to environmental changes. Through a systematic evaluation of 12 species distribution models (SDMs), we ultimately selected the Maximum Entropy (MaxEnt) model for predicting species distributions. The R-optimized MaxEnt model incorporated 11 environmental variables and 218 occurrence records to assess habitat suitability under historical, current, and future climate scenarios (SSP1-2.6 and SSP5-8.5). The model was configured with LQP features and a default regularization multiplier value of 1. Results reveal that temperature (BIO6, BIO2, BIO4) and precipitation (BIO12) are the primary drivers of habitat suitability, with tropical and subtropical regions identified as the most favorable. Future projections indicate a complex pattern of habitat contraction and expansion, with a notable northward shift toward higher latitudes under climate change. These findings highlight the profound impact of climate change on Erythroneurini distribution, underscoring the need for proactive management. Implementing long-term monitoring and targeted control in vulnerable regions mitigates ecological and agricultural risks, supporting sustainable pest management and fostering the integration of ecological conservation with agricultural development.

## Simple Summary

This study utilized an optimized MaxEnt Model to predict the potential distribution of Erythroneurini (Hemiptera: Cicadellidae: Typhlocybinae) in China. The research results indicate that its distribution exhibits an alternating pattern of expansion and contraction, highlighting its adaptability to the environment. The impact of climate change shows characteristics of nonlinear responses, with significant differences observed under different emission scenarios. Notably, the Yangtze River Basin and the North China Plain have become key expansion regions, and it is necessary to establish an adaptive monitoring system. Overall, these research findings have enhanced our understanding of the distribution dynamics of climate-driven species and provided support for adaptive pest management. The research method employed in this study is applicable to other climate-sensitive species, contributing to biodiversity conservation and the sustainable development of agriculture.

## 1. Introduction

Global biodiversity presently faces severe jeopardy, with its root causes in habitat loss and alterations instigated by climate change [1,2,3]. Climate change has exerted multi-dimensional impacts on the biological field, which can change the distribution and migration patterns of species and also affect the structure and function of ecosystems [4,5]. Based on the 3I Interactive Keys and Taxonomic Databases, the subfamily Typhlocybinae (Hemiptera: Cicadellidae) comprises seven valid tribes: Alebrini, Beameranini, Dikraneurini, Empoascini, Erythroneurini, Protodikraneurini, and Typhlocybini (3I; accessed on 15 November 2024; https://hoppers.speciesfile.org/) [6]. Erythroneurini Young, 1952 (Hemiptera: Cicadellidae: Typhlocybinae) is the largest tribe of the subfamily Typhlocybinae and is widely distributed in all major zoogeographic regions of the world, with approximately 200 genera and more than 2000 species currently known [7,8]. In China, more than 450 species belonging to 60 genera of this tribe have been recorded, mainly concentrated in the southern region, which is closely related to the local ecological conditions such as climate and vegetation (3I; accessed on 15 November 2024; https://hoppers.speciesfile.org/) [6,9]. Erythroneurini, characterized by its remarkable species diversity and abundance, plays a significant role in human production and livelihoods [10]. Many species within this tribe are recognized as key agricultural pests, causing substantial economic losses to agriculture, forestry, and animal husbandry through direct damage and disease transmission, posing significant challenges to the agricultural ecosystems of China [11,12,13]. Their impact is multifaceted: for example: *Thaia subrufa* (Hemiptera: Cicadellidae: Erythroneurini) damages rice leaves by sap-sucking, leading to the formation of white spots and streaks, severe desiccation, and impaired photosynthesis, ultimately hindering plant growth [14]. Similarly, *Elbelus tripunctatus* (Hemiptera: Cicadellidae: Erythroneurini) acts as a vector for plant viruses, such as those causing witches’ broom disease in *Melia azedarach* (Meliaceae), exacerbating the spread of plant diseases and further threatening crop health [12,15]. Additionally, *Singapora shinshana* (Hemiptera: Cicadellidae: Erythroneurini) a polyphagous pest, inflicts damage on a wide range of crops, including peach (*Amygdalus persica* L.), hawthorn (*Crataegus pinnatifida* Bge.), apple (*Malus pumila* Mill.), and sweet potato (*Ipomoea batatas* L.), resulting in significant ecological and economic losses [11,16,17]. These species are particularly notable for their high damage potential, rapid reproductive rates, and wide distribution pattern [18], collectively underscoring their substantial and far-reaching impact on human society [10]. Erythroneurini exhibits high sensitivity to climate change, which influences various aspects of its biology, ranging from growth environments to physiological traits [19,20]. Climate change not only disrupts their reproductive cycles [21], but also alters habitat suitability [22,23], and affects access to food resources [24,25]. Furthermore, Erythroneurini represents an ideal model organism with substantial research value for elucidating plant-insect interaction mechanisms, agricultural pest evolution, and organismal responses to climate change [9,10,12,15]. This taxonomic group can function as a bioindicator for assessing environmental quality, biodiversity patterns, and altitudinal/latitudinal gradient variations [15]. It has been successfully employed to monitor ecological restoration processes in karst rocky desertification control regions of southwestern China [9,26,27,28,29]. Additionally, Erythroneurini is an ideal model for biogeographical and phylogeographical research [9]. However, current research on Erythroneurini primarily focuses on taxonomy, phylogeny, and pest control [10,12,30,31,32,33,34]. Despite the pressing challenges posed by climate change, studies on the potential geographical distribution of Erythroneurini remain limited. To date, only Luo et al. (2024) have explored habitat distribution changes of *Seriana bacilla* (Hemiptera: Cicadellidae: Erythroneurini) under different climatic conditions in China [9]. Clearly, a deeper understanding of the tribe’s geographical distribution trends under climate change is essential, necessitating further comprehensive research to inform effective management strategies and mitigate potential ecological and agricultural impacts.

This study focuses on the tribe Erythroneurini to investigate the dynamics of its suitable habitats and the key environmental drivers of climate change. To achieve this, we employ two advanced analytical tools, each selected for its methodological rigor and suitability for addressing specific aspects of the research objectives. The first tool is the MaxEnt model (see the Glossary) [35,36,37], which predicts species distribution based on environmental variables and is well-suited for handling presence-only data and capturing spatial heterogeneity. It has been successfully applied in assessing the diffusion risks of economic pests, such as *Phenacoccus solenopsis* (Hemiptera: Pseudococcidae) [38], *Euplatypus parallelus* (Coleoptera: Curculionidae) [39], *Dalbulus maidis* (Hemiptera: Cicadellidae) [40], *Monochamus alternatus* (Coleoptera: Cerambycidae) [41], *Locusta migratoria* (L.) (Orthoptera) [42], and tribe Zyginellini (Hemiptera: Cicadellidae: Typhlocybinae) [43]. The second tool is the lumped model (see the Glossary), which enhances sample size and correlation analysis by integrating data from closely related species, making it suitable for ecological niche modeling at supra-species levels [44,45,46,47]. Its effectiveness has been demonstrated in studies on the tribe Zyginellini [43], *Osphya* (Coleoptera: Melandryidae) (Beetle Group) [48], and *Limassolla* (Hemiptrea: Cicadellidae: Typhlocybinae) [49], proving its capability to capture distribution patterns and ecological niches at higher taxonomic levels [50]. Moreover, the monophyly of Erythroneurini validates the application of the lumped method [9,51]. By combining these two methods, MaxEnt model provides high-precision predictions, while the lumped model enhances data robustness, collectively revealing the distribution patterns of Erythroneurini and addressing the following three scientific questions: (1) What is the most crucial bioclimatic variable affecting the distribution of Erythroneurini? (2) Under the influence of climate change, will the habitat of Erythroneurini in China contract or expand in the future? (3) In response to climate change, will Erythroneurini migrate northward or to higher latitudes, similar to other insects or animals?

## 2. Materials and Methods

### 2.1. Occurrence Records

**Figure 1:**
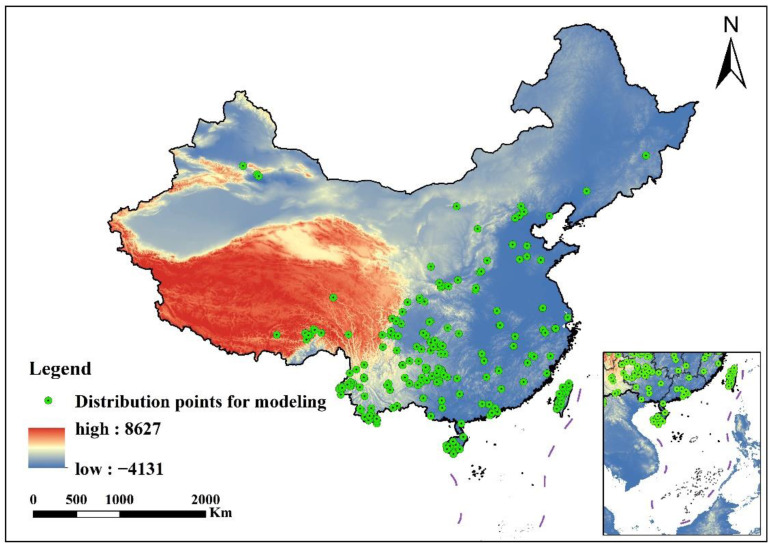
Occurrence points of tribe Erythroneurini.

The integration of taxonomic groups with close genetic relationships through the lumping method facilitates the aggregation of data, enabling a more comprehensive analysis of shared niche requirements and resource utilization patterns, thereby improving the accuracy of niche estimation [44]. In this study, based on the natural monophyly of the tribe Erythroneurini and the similarities in the mitochondrial genome structures of its species, we employed the lumping method to consolidate occurrence data from diverse species within Erythroneurini, generating a unified dataset that encapsulates the entire tribe [9,33]. Occurrence data for Erythroneurini in China were sourced from multiple publicly accessible databases: (1) 3I Interactive Keys and Taxonomic Databases, an online repository of interactive identification keys and taxonomic information (3I; accessed on 15 June 2024; https://hoppers.speciesfile.org/) [6]; (2) Global Biodiversity Information Facility (GBIF; accessed on 18 June, 2024; https://www.gbif.org/); (3) academic monographs, including Erythroneurini and Zyginellini from China (Hemiptera: Cicadellidae: Typhlocybinae) and A Taxonomic Study of Chinese Cicadellidae (Hemiptera), along with several related zoological publications [9,10,12,15]; (4) Our research team has collected and documented field specimens from approximately 300 distinct geographical locations since 2019 [9,27,28,29,52,53,54,55]. The original dataset comprised 940 occurrence records across 59 genera. It is critical to acknowledge that uneven sampling efforts across regions can introduce sampling bias and overfitting, potentially compromising the transferability of niche models [56,57,58]. To address these issues, we curated distribution data with precise longitude and latitude coordinates, removed duplicate and erroneous entries, and supplemented missing records using the coordinate picker tool from the Gaode Open Platform (accessed on 25 June 2024; https://lbs.amap.com/tools/picker). To minimize the effects of spatial autocorrelation, we applied a 2.5 km buffer using ArcGIS 10.4.1 (ESRI Inc, Redlands, CA, USA), consistent with the spatial resolution of environmental data. This process resulted in 218 validated occurrence records, encompassing 59 genera and 168 representative species. The data were formatted in “csv” files, organized by name, longitude, and latitude (Table S1), and imported into ArcGIS. After standardizing the spatial reference to WGS84, the data were exported for subsequent model analysis (Figure 1). The implementation of the aforementioned procedures effectively guarantees the establishment of a reliable and representative dataset, significantly enhancing the quality of the samples [56,58].

### 2.2. Acquisition and Organization of Environmental Data

#### 2.2.1. Data Acquisition

Species distribution is primarily influenced by climatic factors, such as temperature, humidity, precipitation, and accumulated temperature, at relatively large scales [57,59]. At finer geographical scales, however, biotic interactions, dispersal capacity, and anthropogenic disturbances exert equally significant influences on distribution pattern formation [35,57]. Therefore, the selection of environmental variables must comprehensively consider their biological significance and geographical relevance to ensure accurate modeling [57]. Global Climate Models (GCMs) (see the Glossary) provide a robust framework for simulating the behavior and dynamics of the Earth’s climate system, enabling predictions of both future and past climate trends [60]. In this study, a set of bioclimatic raster data variables (Bio1–Bio19) with a spatial resolution of 2.5 arc-min (approximately 5 km^2^ at ground level) was obtained from the WorldClim Global Climate Data website (https://worldclim.org/, accessed on 20 July 2024). These data encompass historical periods (Last Glacial Maximum [LGM, approximately 22,000 years ago] and Mid-Holocene [MH, approximately 6000 years ago]), the current period (1970–2000 average), and future climate scenarios based on the BCC-CSM2-MR climate model [61]. The 19 bioclimatic variables capture annual variations, seasonal characteristics, and extreme conditions in both temporal and spatial dimensions, offering enhanced biological significance for ecological modeling [60,62]. For future climate projections, the data are derived from the downscaled outputs of the Coupled Model Intercomparison Project Phase 6 (CMIP6), which better reflects the interplay between climate change and socioeconomic development. These projections include three time periods (2050s, 2070s, and 2090s) under the Shared Socioeconomic Pathways (SSPs) scenarios SSP1-2.6 and SSP5-8.5 [3,63,64,65]. The SSP1-2.6 scenario represents a sustainable development pathway, emphasizing environmental protection and sustainable resource utilization, with low greenhouse gas emissions and a stabilized radiative forcing of approximately 2.6 W/m^2^. This scenario results in a relatively stable global climate system with minimal climate change impacts [5]. Conversely, the SSP5-8.5 scenario represents a high-emission pathway, with radiative forcing potentially reaching 8.5 W/m^2^, leading to drastic climate changes, including significant temperature increases and pronounced shifts in precipitation patterns [5]. The BCC-CSM2-MR climate model has demonstrated its ability to accurately reproduce climate distribution characteristics and exhibits high predictive accuracy, particularly in assessing and projecting climate change impacts in China [65].

Topographic factors, including elevation, slope gradient, and aspect, indirectly influence organisms by modulating key climatic factors such as temperature, precipitation, and sunlight. Elevation data, with a spatial resolution of 2.5 arc-min, were obtained from the World Climate Database. Slope and aspect were subsequently derived from the elevation data using the Raster Surface tool in ArcGIS. Additionally, the Normalized Difference Vegetation Index (NDVI), a critical indicator of vegetation cover and health, was sourced from the Resource and Environment Science Data Registration and Publishing System (accessed on 21 July 2024; http://www.resdc.cn/). To simplify the complex environmental system in this study, we adopted a relatively static assumption, positing that topographic factors and biological variables would remain stable and not undergo significant changes in the future [66,67]. This assumption allowed us to apply these variables consistently across prediction models for different time periods [68]. To ensure consistency and compatibility across datasets, all raster data were preprocessed to align with the WGS84 coordinate system, and the spatial resolution of environmental parameters was standardized to 2.5 arc-min. The processed raster dataset was then converted to the “asc” format using the Raster to ASCII (Folder) function within the SDM Toolbox v2.6 [69].

#### 2.2.2. Data Preprocessing

The future climate data comprise a multi-band image containing 19 individual bands. To process this data, the downloaded GeoTiff files were imported into ArcGIS, and a series of operations were performed using the SDM Toolbox v2.6 and Data Management Tools. These operations included “Define Projection as WGS84 (Folder)”, “Resample Grids (Folder)—Nearest Neighbor”, “Extract Bands”, “Extract By Mask (Folder)”, and “Raster to ASCII (Folder)”. The mask data, an “eps” format file, was obtained from the Standard Map Service System of the Ministry of Natural Resources (accessed on 11 July 2024; http://bzdt.ch.mnr.gov.cn/), with the review number GS (2024) 0650. After removing the filled color and saving the map with the WGS84 spatial reference, no further modifications were made to the map in subsequent procedures. Multicollinearity among environmental factors can compromise the model’s ability to accurately assess the contribution of each factor to the prediction outcomes, leading to instability and reduced accuracy [70,71]. To address this issue, a systematic approach was adopted, including the construction of a basic model framework and variable screening. First, the current 23 environmental variables and the filtered 218 distribution data were imported into MaxEnt v3.4.1. The model was configured with default parameters, and the distribution data were partitioned into quarters, with 75% used as the training dataset and the remaining 25% reserved for model validation. Background points were set at 10,000, and the algorithm underwent 5000 iterations to ensure sufficient convergence time [72]. The process was repeated 10 times using the “Bootstrap” method, while other settings remained at default values. The prediction results were output in Logistic format and ASC file types. The relative contribution of each environmental variable was evaluated through the percent contribution rate, permutation importance, and the Jackknife test [73]. To mitigate multicollinearity, Pearson correlation analysis was conducted on the 23 predictor variables using the “corrplot” function in R 4.3.2 (Figure S1a) [74]. To prevent model overfitting, the contribution of environmental variables and the biological characteristics of Erythroneurini were comprehensively considered. Within each variable group where the correlation coefficient r ≥ |0.8|, the variable with a lower contribution rate or less biological significance was excluded. Ultimately, 11 environmental variables were selected for model optimization (Figure S1b; Table 1) [75].

### 2.3. Model Optimization, Construction, and Accuracy Assessment

In this study, the “sdm” package in R software was employed to evaluate 12 distinct models for their suitability in predicting species distribution (Figure S2) [76]. Model performance was assessed using three key metrics: (1) the Area Under the Receiver Operating Characteristic Curve (AUC), which quantifies the model’s ability to discriminate between presence and absence records [77]; (2) the True Skill Statistics (TSS), which provides a comprehensive measure of prediction accuracy [42,78]; and (3) the difference between training AUC and testing AUC (avg.AUC_DIFF_), which evaluates the degree of overfitting and the model’s generalization capability [35,79]. Based on the comprehensive evaluation results (Table S2), the MaxEnt model was selected as the optimal choice due to its superior performance [76].

In this study, MaxEnt v3.4.1 was selected as the modeling tool, and the R packages “ENMeval v2.0.4” [80] and “dismo v1.3.5” [81] were employed to optimize the regularized multipliers (RM) (see the Glossary) and feature combinations (FC) (see the Glossary), aiming to reduce model complexity and enhance prediction accuracy [66,80,82]. A total of 56 parameter combinations were generated by integrating seven FCs (L, LQ, H, LQH, LQP, LQHP, LQHPT) and eight RMs (ranging from 0.5 to 4.0 at intervals of 0.5) [83]. The distribution data were partitioned using the Checkerboard 2 method [80], a geographic structuring approach that effectively adjusts the regularization level of the model. The Akaike Information Criterion corrected (AICc) was applied to assess the complexity and goodness of fit of different parameter combinations, with the combination yielding the lowest AICc value (delta.AICc = 0) selected for final modeling [84]. When the model was constructed using default parameters (RM = 1 and FC = LQPHT), the delta.AICc value was 106.07, indicating significant overfitting. However, when optimized parameters (FC = LQP and RM = 1) were applied, the delta.AICc value dropped to 0 (Figure S3, Table S3). Under the optimized configuration, the avg.AUC_DIFF_ and or.10pct values were 0.0196 and 0.1519, respectively, representing reductions of 57.02% and 26.94% compared to the default settings. This demonstrates a substantial improvement in model performance and robustness.

The AUC value, a widely used and threshold-independent evaluation metric, measures the model’s ability to distinguish between species presence and absence [50,85], thereby evaluating its generalization ability and stability of the model [86]. The AUC value ranges from 0 to 1, with values closer to 1 indicating higher model credibility [87]. The following classification is generally accepted: 0–0.5 (fail), 0.5–0.7 (poor), 0.7–0.8 (fair), 0.8–0.9 (good), and 0.9–1.0 (excellent) [85]. The optimized MaxEnt model in this study achieved an AUC value within the “excellent” range, underscoring its reliability and predictive accuracy.

### 2.4. Division of Suitable Distribution Area

Firstly, the average output results from 10 runs of the MaxEnt model were imported into ArcGIS for visualization. The suitable habitats were classified into distinct grades using the Maximum Test Sensitivity Plus Specificity Logistic threshold (MTSPS) (see Glossary) [88]. Based on the MTSPS value of 0.22 and in alignment with the species distribution pattern, the suitable habitat area for Erythroneurini was categorized into four grades: (1) non-suitable area (*p* < 0.22), (2) low-suitable area (0.22 ≤ *p* < 0.44), (3) medium-suitable area (0.44 ≤ *p* < 0.66), and (4) high-suitable area (*p* ≥ 0.66). Here, *p* denotes the suitability probability, ranging from [0, 1].

### 2.5. Dynamic Change in Suitable Areas

**Figure 2:**
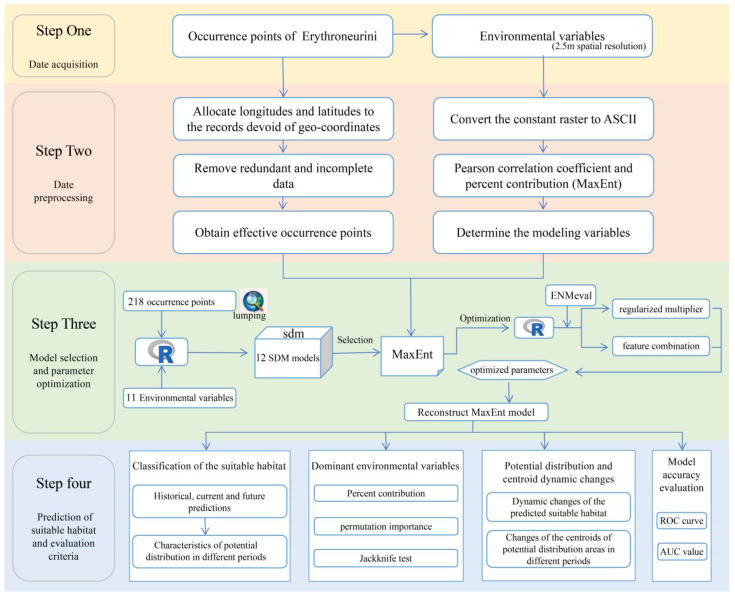
Flowchart presents the steps of the present study and shows the sequential processes and activities involved in the research.

To explore the potential changes in the distribution areas of Erythroneurini under different climate scenarios across various time periods, a systematic and multi-step analytical approach was employed. First, suitable and non-suitable areas were delineated based on the Habitat Suitability Index (HIS) (see the Glossary) derived from the MaxEnt model output [89]. Using the Quick Reclassify to Binary in the SDM Toolbox v2.6, a binary raster file was generated, classifying areas as either potentially suitable (HSI > MTSPS, defined as 1) or non-suitable (HSI < MTSPS, defined as 0), among them, MTSPS = 0.22 [90]. Next, the HSI was utilized to analyze the proportional distribution of Erythroneurini across different habitat suitability levels. The proportion of spatial distribution quantifies the relative distribution of a species across areas of varying suitability levels [91]. The calculation formula is as follows: The distribution proportion of a certain suitability level area = (The number or area of species distribution in this area/The total number or area of species distribution) × 100%. Then, based on the unrestricted migration assumption [47], the binary maps of non-suitable and potentially suitable areas for each period were generated, with values defined as 0 for no occupancy (absence in both periods), −1 for range expansion, 1 for no change (presence in both periods), and 2 for range contraction. After modeling the suitable habitats of Erythroneurini, the resulting matrix file was imported into ArcGIS to compute changes (expansion, stability, and contraction) in suitable areas compared to the current period. The geometric center position (centroid) of the species’ suitable area was calculated to represent its overall spatial position, and the shift trend and distance of the centroid were used to characterize the overall migration trend and distance of Erythroneurini’s suitable area in China. Our study aims to show the research ideas and research steps in Figure 2 (presented briefly).

## 3. Results

### 3.1. Analysis of Dominant Factors in Geographic Distribution Patterns of Erythroneurini

To assess the training effectiveness of the model and the relative importance of environmental variables, we utilized Regularized Training Gain (RTG) (see the Glossary) and Percent Contribution (PC) (see the Glossary), respectively [36]. The Jackknife test results indicated that, when evaluated individually, BIO6 (RTG = 0.98, PC = 28.1%), BIO4 (RTG = 0.84, PC = 19.3%), BIO12 (RTG = 0.81, PC = 2.7%), and BIO2 (RTG = 0.71, PC = 21.7%) were the dominant factors influencing the spatial distribution of Erythroneurini (Table 1; Figure S4). In addition, BIO14, NDVI, and BIO15 were identified as significant contributors to the spatial distribution of Erythroneurini. To further explore the relationship between habitat suitability and environmental factors, we analyzed and plotted the response curves of the four dominant factors using logistic regression within the MaxEnt model. Based on these analyses, areas with an HSI exceeding 0.22 were deemed suitable for Erythroneurini survival. Specifically, the species was found to thrive in regions where BIO6 was greater than −8.75 °C, BIO12 was greater than 667.84 mm, BIO4 was less than 882.12, and BIO2 was less than 11.71 °C. Notably, the occurrence probability peaked when BIO6 was 17.79 °C, BIO12 was 4017.41 mm, BIO4 was 277.47, and BIO2 was 4.64 °C (Figure S5). These findings highlight that low-temperature stress, environmental temperature stability, precipitation amounts, and the seasonal variation of precipitation are critical determinants of Erythroneurini’s growth and spatial distribution, with its distribution shaped by the combined effects of these key climatic factors. Field observations by our research team corroborate these results, as Erythroneurini, being a poikilothermic organism, exhibits active reproductive behavior during warm and humid seasons, while its population significantly declines during cold and dry periods. The strong alignment between model predictions and observed biological characteristics further validates the reliability of the findings and underscores the profound influence of climatic factors on the species’ ecological dynamics [9,26].

### 3.2. Current Potential Suitable Areas of Erythroneurini in China

**Figure 3:**
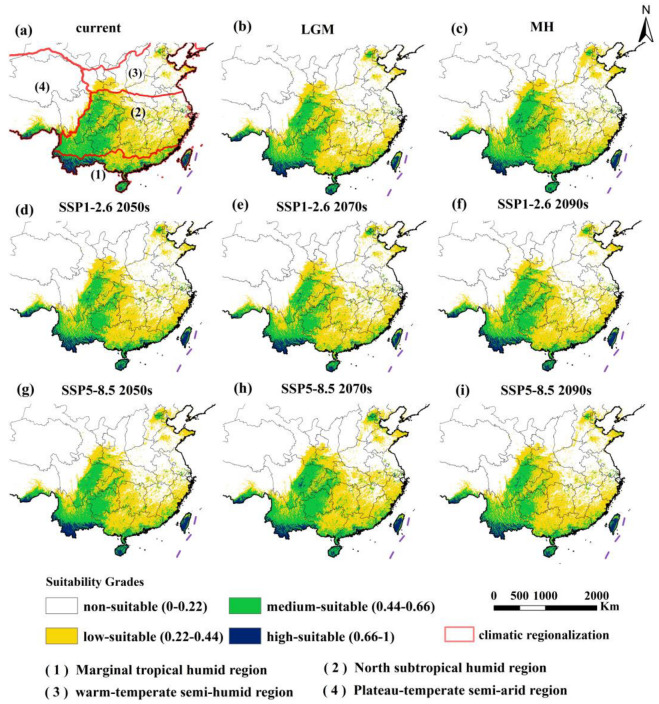
Potential distribution areas of tribe Erythroneurini under different climate scenarios. Note: The red lines are the climatic regionalization. Note: The climate regions divided by the red lines include: (1) the marginal tropical humid regions, (2) the northern subtropical humid regions, (3) the warm temperate semi-humid regions, and (4) the plateau temperate semi-arid regions.

For the 218 distribution points of the tribe Erythroneurini under the contemporary climate scenario, the HSI values for high-suitable areas, medium-suitable areas, low-suitable areas, and non-suitable areas were determined to be 0.23, 0.44, 0.21, and 0.11, respectively. The majority of the sample points were located within the suitable habitat area, strongly validating the model’s effectiveness in predicting the potential spatial distribution of Erythroneurini. The total area of suitable habitat for Erythroneurini in China during the current period was approximately 2.12 × 10^6^ km^2^ (Table 2), accounting for about 22.07% of the total land area of China. The medium-suitable areas, covering 7.98 × 10^5^ km^2^, were predominantly distributed in central-northern Yunnan, western Guizhou, eastern Sichuan, southern Guangxi, and Guangdong, most of Hainan, Taiwan, and southeastern Tibet (Figure 3a). These regions are characterized by favorable climatic conditions, including moderate temperatures and sufficient precipitation. The high-suitable areas, with a total area of 1.34 × 10^5^ km^2^, were either adjacent to or interspersed with the medium-suitable areas, primarily located in southern Yunnan, Taiwan, southern Tibet, central Guangdong, central Hainan, and sporadically in southwestern Guangxi, eastern Sichuan, and western-eastern Guizhou. These areas are distinguished by their warm and humid climates, particularly in coastal regions, which provide optimal conditions for Erythroneurini survival and reproduction. Overall, under the current climate scenario, the suitable habitats of Erythroneurini were mainly located south of the 400 mm equivalent precipitation line and concentrated in regions south of the Qinling-Huaihe line and north of the Yangtze River basin. In terms of climate zones, the medium-suitable areas were predominantly distributed in the southern portion of the marginal tropical humid zone and the western section of the northern subtropical humid zone, as well as in the southern part of the plateau temperate semi-arid region and the northeastern part of the warm temperate semi-humid region. The high-suitable areas were mainly concentrated in the marginal tropical humid zone, particularly in coastal regions characterized by warm and humid climates.

### 3.3. Spatial Distribution of Erythroneurini in Different Periods

Biogeographical and paleogeographical evidence strongly suggests that the Erythroneurini tribe originated from Laurasia during the Pangaea era [92]. Molecular clock analysis indicates its divergence occurred around 85.52 million years ago, after which the group diversified and dispersed across regions in response to environmental and tectonic changes [9,92]. In the LGM climate scenario, Erythroneurini was mainly distributed in the area south of the Qinling-Huaihe line and east of the Hengduan Mountains, including marginal tropical humid regions, northern subtropical humid regions, plateau temperate semi-arid regions, and warm temperate semi-humid regions. The highly suitable areas were located in the hilly areas of the river valleys in southern Yunnan, the southern flank of the Eastern Himalayas, the lowlands and hills of the Qionglei Peninsula, and the northern and southern lowlands of Taiwan Province (Figure 3b)., It can be seen that during the MH period, most of southeastern and southwestern China, as well as the Liaodong Peninsula, were suitable areas (Figure 3c). Overall, under the SSP1-2.6 climate scenario, from the LGM to the 2090s, the change in the area of suitable habitats exhibited a rise-then-fall trend. With a maximum change rate of −6.67%, it peaked at 213.16 × 10^4^ km^2^ during the MH. Conversely, the high-forcing SSP5-8.5 scenario resulted in a more complex pattern, with the maximum change rate reaching 13.80%. It started to decline after reaching its peak in the 2070s. The area in the 2070s increased by 6.21% compared to the current era (Table 3). Specifically, under the SSP5-8.5 scenario, the area of High-suitable habitats for Erythroneurini peaked at 13.61 × 10^4^ km^2^ in the 2070s, and then decreased to 11.70 × 10^4^ km^2^. This decline strongly indicates that extreme high-temperature events associated with climate change could undermine the long-term viability of these highly suitable regions. During the same period, the area of moderately suitable habitats first increased to 86.81 × 10^4^ km^2^ in the 2070s, but then dropped to 66.20 × 10^4^ km^2^ afterwards. The relatively larger magnitude of change in the Medium-suitable habitats suggests that these areas are more vulnerable to the impacts of climate fluctuations. Overall, compared with the low-forcing climate scenario (SSP1-2.6), the total area of potentially suitable habitats under the high-forcing climate scenario (SSP5-8.5) exhibited an upward trend. These results highlight the significant impacts of different climate change conditions on the distribution of suitable habitats for Erythroneurini.

### 3.4. Potential Distribution and Centroid Dynamic Changes

**Figure 4:**
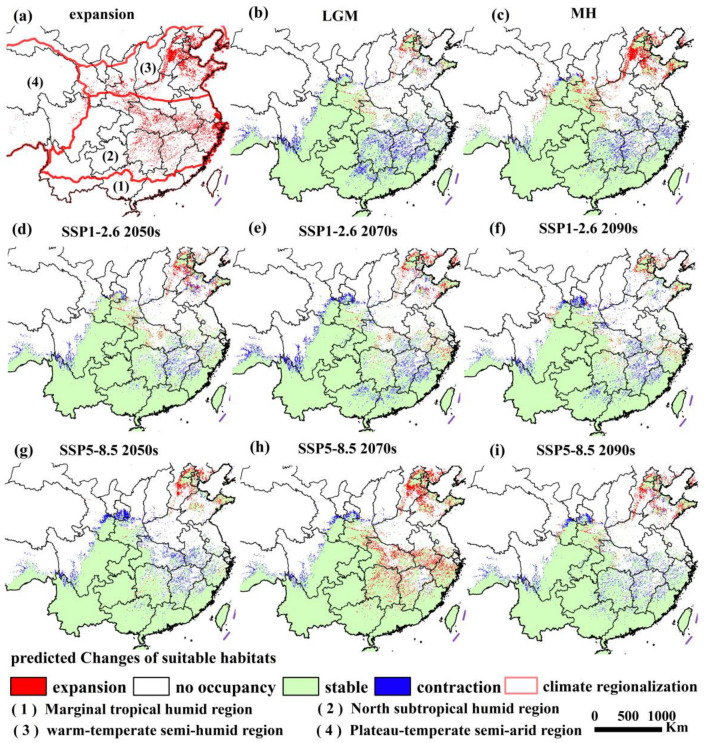
Dynamic changes of the potentially suitable habitats of Erythroneurini compared to the current period under different climate scenarios. The regions divided by the red lines are the same as those in Figure 3.

**Figure 5:**
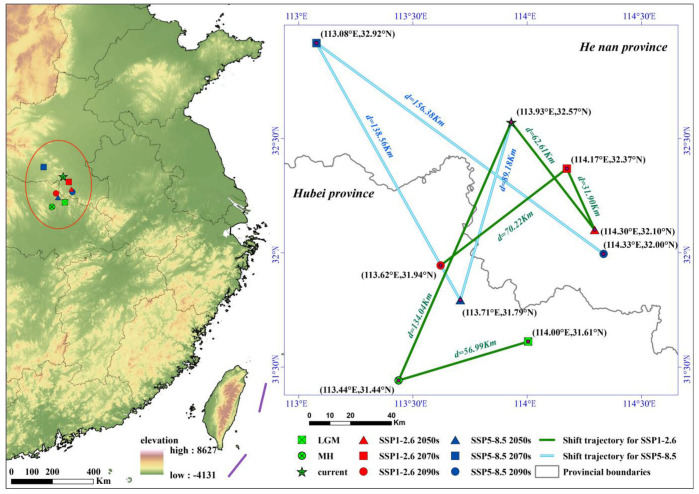
Distribution centroid shift dynamics of Erythroneurini.

We took the current distribution as a benchmark to calculate the expansion or contraction situations in different periods. We superimposed and analyzed all expansion results to obtain the overall expansion situation of Erythroneurini (Figure 4a). Areas such as the North China Plain, Liaodong Peninsula, Hanzhong Basin, the middle and lower reaches of the Yangtze River Plain, and Jiangnan hilly region will be the main expansion regions for Erythroneurini in the future, and the range of their suitable habitats will expand to higher latitudes to a certain extent (Figure 4b–i). The warm and humid climate as well as the abundant vegetation resources in these regions provide a suitable living environment for Erythroneurini. Compared with the MH, the current centroid has shifted towards the northwest, and the shift distance is 134.04 km (Figure 5, Table S4). Under the future SSP1-2.6 2070s, the centroid continued to shift towards the northwest in comparison to that in the 2050s, with a shift distance of 31.90 km and a shift speed of 1.60 km yr^−1^. Under the scenario of SSP5-8.5 in the 2070s, with the intensification of warming, it continued to shift towards the northwest at a higher latitude from the 2050s by 138.56 km, with a shift speed of 6.93 km yr^−1^. In general, both the shift distance and the shift speed of the centroid under the SSP5-8.5 climate scenario exceed those under the SSP1-2.6 climate scenario, thereby indicating that different climate scenarios exert varying degrees of influence on the species’ spatial distribution. Meanwhile, although various changes, such as alterations in temperature and precipitation patterns, have occurred from the LGM to different future periods, the centroid has always been stably located within the northern subtropical humid climate zone of China.

## 4. Discussion and Conclusions

The rapid acceleration of global climate change has given rise to remarkable and persistent alterations in crucial climatic factors such as temperature, precipitation, and sunshine duration [23,93]. Recent research leveraging niche modeling and climate scenario simulations has revealed that, in the face of these climate alterations, leafhopper species are experiencing profound transformations in their habitat selection preferences and potential distribution patterns [9,40,43,49,94]. Understanding the evolution of these distribution patterns under climate change is critical for developing effective strategies for species adaptation, ecological conservation, and pest management [41,75].

To meet the requirements for model applicability and accuracy, this study conducted a systematic evaluation of 12 commonly used SDMs and innovatively selected the MaxEnt model to predict potentially suitable habitats and spatial distribution patterns of Erythroneurini. Through parameter optimization, the predictive accuracy and transferability of the MaxEnt model were significantly improved [88]. By integrating Geographic Information Systems (GIS), big data analytics, and machine learning algorithms, the study evaluates habitat suitability and predicts dispersal pathways under historical, current, and future climate scenarios (SSP1-2.6 and SSP5-8.5) [42]. The results demonstrate that the distribution patterns of Erythroneurini are primarily regulated by temperature factors (low-temperature stress and temperature stability) and precipitation factors (precipitation amount and its seasonal variations). Additionally, the inherently limited dispersal capacity of Erythroneurini serves as another critical factor constraining its distributional dynamics.

Under climate change, the suitable distribution range of Erythroneurini exhibits a dual trend of expansion and contraction. Compared to the current suitable habitat area, the distribution range contracted significantly (−6.67%) during the LGM but expanded slightly (+0.55%) during the MH. Under the SSP1-2.6 scenario, contraction predominates (2050s: −1.59%; 2070s: −4.22%; 2090s: −3.87%). In contrast, the SSP5-8.5 scenario shows a nonlinear response pattern (2050s: −3.83%; 2070s: +6.21%; 2090s: −2.98%), potentially associated with extreme climate events. For instance, rising temperatures may reduce habitat suitability in later periods. Notably, southwestern China, Taiwan, and Hainan have been identified as potential biodiversity hotspots for the tribe Erythroneurini. Our research team’s 2021 field surveys further confirmed that the karst regions of southwestern China (including Yunnan, Sichuan, Guangxi, Guizhou, and Chongqing provinces) constitute the primary distribution area of Erythroneurini, while Hainan and Taiwan serve as species-rich distribution hotspot centers for these leafhoppers [9]. Under future climate scenarios, the tribe exhibits a northward expansion trend [1]. The Yangtze River Basin provinces (Anhui, Hubei, Zhejiang, and Jiangxi) and the North China Plain have emerged as potential expansion frontiers for Erythroneurini. The northward range expansion of these leafhoppers may exert profound impacts on local agroecosystems. Therefore, implementing adaptive monitoring systems in these regions is of critical importance.

From the perspective of pest management, the predicted habitat suitability results provide critical insights for identifying key prevention and control zones. By integrating the biological characteristics and occurrence patterns of Erythroneuri species, researchers can develop and implement Integrated Pest Management (IPM) strategies [95,96]. In high-risk areas, establishing a “trinity” prevention and control system (real-time monitoring network + natural enemy conservation system + precision pesticide application technology) can effectively regulate pest populations [41]. For regions projected to experience pest range expansion, such as the North China Plain and the Liaodong Peninsula, the establishment of regional monitoring and early warning systems is essential [13]. Advanced monitoring networks can track population density dynamics and occurrence patterns, enabling timely and targeted intervention measures [97].

In practical applications, predictive models (particularly climate models and ecological niche models) inevitably exhibit varying degrees of uncertainty. This uncertainty primarily stems from the following aspects: measurement errors in environmental variables, incompleteness of species distribution data, and inherent limitations of the modeling algorithms (including the validity of fundamental assumptions, accuracy of parameterization schemes, and complexity of computational algorithms) [35,36,57,67,80]. Despite the successful application of the optimized MaxEnt model in predicting the potential suitable distribution areas of Erythroneurini in China, certain limitations remain. First, reliance on a single model may oversimplify the complexity of species distribution dynamics, potentially leading to partial or biased prediction results. Second, compared to integrated modeling approaches developed for individual species, the lumping method faces challenges in accurately analyzing each species’ unique contribution to the overall distribution pattern and quantifying relevant error factors [35,44,82]. Even if the Erythroneurini leafhoppers form a monophyletic group, their actual distribution may deviate from predictions generated by the lumping method due to interspecific variations in microhabitat differentiation and host plant preferences. This limitation may obscure species-specific responses to environmental changes [44]. Additionally, the real-world ecosystem is influenced by a multitude of factors, including host crop distribution and human activities such as land-use changes and agricultural practices, and other human activities, which were not fully accounted for in this study [35,40]. Therefore, future research should adopt more comprehensive and integrated approaches to address these limitations in ecological studies [35,66,67].

In this study, although the MaxEnt model optimized by ENMeval has improved its predictive accuracy, due to the inherent limitations of the algorithm and the uncertainties of climate scenarios [35], the potentially suitable distribution areas predicted by the model are only similar to the environmental conditions of the current distribution areas [57,58,75,80]. Therefore, the output results should be regarded as “possible distribution areas” rather than the exact actual distribution [37,57]. Moreover, the complexity of the real-world ecosystem far exceeds what the model can take into account [35,98]. The distribution of host crops, changes in land-use patterns, agricultural practices, and many other environmental and anthropogenic factors all have an impact on the actual distribution of the tribe Erythroneurini [40]. However, due to the limitations of the model’s own design and research conditions, it is impossible to comprehensively incorporate all these influencing factors into the analysis [40,50]. To address these limitations, future research should prioritize multi-model integration and the comparison of multiple climate models to reduce uncertainties and enhance predictive accuracy [43,45,46]. Additionally, incorporating both biotic factors (e.g., host plant interactions) and abiotic factors (e.g., climate and soil conditions) [40,57], along with targeted analyses of individual species [44], will provide a more comprehensive understanding of the habitat selection and population dynamics of Erythroneurini. These advancements will improve the robustness of predictions and support more effective conservation and pest management strategies.

## Figures and Tables

**Table 1:** Environmental variables used in this study to model potential distribution of tribe Erythroneurini.

Variables ^a^/Description	Percent Contribution/%	Permutation Importance/%	Regularized Training Gain
Min Temperature of Coldest Month (BIO6/°C)	28.1	21.4	0.98
Mean Diurnal Range (BIO2/°C)	21.7	2.7	0.71
Temperature Seasonality (SD × 100) (BIO4)	19.3	41.7	0.84
Isothermality (BIO2/BIO7) (×100) (BIO3)	10.6	5.6	0.20
Ground height above sea level (Elevation/meter)	4.2	8.2	0.18
Normalized Difference Vegetation Index (NDVI)	4.1	7.7	0.33
Precipitation Seasonality (BIO15/mm)	2.9	3.4	0.20
Annual Precipitation (BIO12/mm)	2.7	3.1	0.81
Slope/Degrees	2.4	1.9	0.02
Precipitation of Driest Month (BIO14/mm)	2	2.7	0.33
Aspect/Degrees	1.9	1.5	0.03

^a^ the table shows the environmental variables used for the final modeling. Data source: WorldClim v2.1-Bioclimatic variables (https://worldclim.org/, accessed on 20 July 2024); Resource and Environment Data Cloud Platform-NDVI (https://www.resdc.cn/, accessed on 20 July 2024).

**Table 2:** The suitable areas of tribe Erythroneurini in China under different climate conditions. The unit of the area is 10^4^ km^2^.

	Historical Climate Scenarios			Future Climate Scenarios					
Classification Level	Current	LGM	MH	SSP126 2050s	SSP585 2050s	SSP126 2070s	SSP585 2070s	SSP126 2090s	SSP585 2090s
None-suitable	748.56	762.70	747.40	751.94	756.68	757.51	735.39	756.77	754.88
Low-suitable	118.80	117.77	119.44	127.46	117.31	124.68	124.76	120.54	127.78
Medium-suitable	79.82	67.65	81.24	68.09	73.19	66.45	86.81	70.11	66.20
High-suitable	13.37	12.44	12.48	13.08	13.38	11.93	13.61	13.14	11.70
Total-suitable	212.00	197.86	213.16	208.62	203.88	203.05	225.17	203.79	205.68

**Table 3:** Dynamic changes of the potential suitable habitats of Erythroneurini under different climate scenarios, compared to the current period. The unit of the area is 10^4^ km^2^.

Period	Area	Gain	Stable	Loss	Range Change (%)	Percentage Loss (%)	Percentage Gain (%)
current	212	/	/	/	/	/	/
LGM	197.86	3.64	211.84	19.27	−6.67	9.09	1.72
MH	213.16	12.18	219.08	12.02	+0.55	5.67	5.74
SSP126 2050s	208.62	6.33	220.69	10.41	−1.59	4.91	2.99
SSP126 2070s	203.05	4.53	215.17	15.94	−4.22	7.52	2.14
SSP126 2090s	203.79	4.53	217.66	13.45	−3.87	6.34	2.14
SSP585 2050s	203.88	4.63	217.69	13.42	−3.83	6.33	2.18
SSP585 2070s	225.17	18.76	225.92	5.19	+6.21	2.45	8.85
SSP585 2090s	205.68	5.55	218.33	12.78	−2.98	6.03	2.62

## Data Availability

The original contributions presented in the study are included in the article/Supplementary Materials, further inquiries can be directed to the corresponding author.

## Supplementary Materials

The following supporting information can be downloaded at https://www.mdpi.com/article/10.3390/insects16050450/s1, Figure S1a: Pearson correlation analysis among the 23 environmental variables. Figure S1b: Multicollinearity test of Pearson correlation analysis between environmental variables retained for MaxEnt modeling. Figure S2: The receiver operating characteristic (ROC) curves of the 12 species distribution models. Figure S3: Results of three evaluation metrics of 56 MaxEnt models run using different combinations of regularization multiplier and feature combination. Figure S4: The relative importance of different predictor variables based on the results of the jackknife test in MaxEnt. Figure S5: Relationships between dominant environmental variables and suitability probability of tribe Erythroneurini. Table S1: The distribution points of the tribe Erythroneurini for modeling. Table S2: Comparison of evaluation criteria for 12 species distribution models. The bold font represented the final prediction model. Table S3: Evaluation metrics of the MaxEnt model generated by ENMeval. Table S4: Centroid distributional shifts under different climate scenarios/periods for the tribe Erythroneurini.

## Glossary

MaxEnt Model (Maximum Entropy Model)	The MaxEnt Model is a methodology that utilizes statistics and related algorithms. It constructs models based on the known data of species occurrences and relevant environmental factors to predict the potential distribution of species under different spatiotemporal conditions. Moreover, it serves as an important tool for examining the habitat suitability of species and carrying out pest management.
Lumping Method	For the occurrence points of species with related lineages or similar ecological requirements, they can be treated as a whole. By integrating the information on these distribution points, the common ecological characteristics among species can be highlighted more effectively.
Lumped Model	The lumped model is a type of model that simplifies complex systems. It assumes that all parts within the system are uniform. It regards the system as a whole and ignores the detailed differences and spatial distribution within the system.
GCMs (Global climate models)	GCMs are powerful tools that provide us with quantitative and scientific bases for understanding past, present, and future climate conditions, as well as for assessing the interaction between climate and human activities. They have far-reaching impacts on various aspects such as the sustainable development of human society, ecosystem protection, and responding to climate change.
FC (feature combinations)	include linear (L), quadratic (Q), product (P), threshold (T), and hinge (H).
PC (Percent contribution)	It is used to measure the relative contribution degree of each feature (variable) to the model’s prediction results.
RTG (Regularized Training Gain)	It refers to the gain achieved by the model during the training process after regularization treatment. The training gain reflects the degree of improvement in the model’s performance on the training data.
MTSPS	Maximum test sensitivity plus specificity Logistic threshold. The optimal threshold found through MTSPS enables the model to achieve better performance on both the training data and the test data. It can enhance the generalization ability and stability of the model, thereby strengthening the accuracy and reliability of predictions.
HSI (Habitat Suitability Index)	HSI is a quantitative indicator that comprehensively evaluates the suitability level of a habitat for a species. Its value range is generally from 0.0 to 1.0. A value of 0.0 represents an unsuitable habitat, meaning that the habitat can hardly meet the basic needs of species for survival, reproduction, and so on. While a value of 1.0 indicates the most suitable habitat, that is, all kinds of environmental conditions in this habitat are quite ideal and can support the survival and reproduction of species to the greatest extent.
