# Supplementary material for: Potential Distribution of Tribe Erythroneurini in China Based on the R-Optimized MaxEnt Model, with Implications for Management
Source: Insects. 2025 Apr 24;16(5):450. doi: 10.3390/insects16050450 (PMC12112222; doi:10.3390/insects16050450)
Supplement: Supplementary file 1 [file insects-16-00450-s001.zip › insects-3583766-supplementary.pdf]

Supplementary Materials

Potential Distribution of Tribe Erythroneurini in China Based on the R-Optimized MaxEnt Model, with Implications for Management

Xiaojuan Yuan [1,2], Weiwei Ran [1,2], Wenming Xu [1,2], Yuanqi Zhao [1,2], Di Su [1,2] and Yuehua Song [1,2,*]

[1] School of Karst Science, Guizhou Normal University, Guiyang 550025, China
[2] State Engineering Technology Institute for Karst Desertification Control, Guiyang 550025, China
* Correspondence: songyuehua@163.com

Contents

Page S1-S6

Table S1. The distribution points of tribe Erythroneurini for modeling. Note: the distribution data of Erythroneurini screened out through steps such as collection, correction, and sparsification.

Page S7

Figure S1 (a) Pearson correlation analysis among the 23 environmental variables. **(b)** Multicollinearity test of Pearson correlation analysis between environmental variables retained for MaxEnt modeling. Note: Environmental variables with Pearson correlation coefficient less than |0.8| were retained for the next step of operation.

Page S8

Figure S2 The receiver operating characteristic (ROC) curves of the 12 species distribution models: (A) Domain; (B) Classification and Regression Trees (CART); (C) Boosted Regression Trees; (D) Multivariate Adaptive Regression Spline (MARS); (E) Maxlike; (F) Bioclim; (G) Generalized Linear Model (GLM); (H) Generalized Additive Model (GAM); (I) Mixture Discriminant Analysis (MAD); (J) Random Forests (RF); (K) Support Vector Machine (SVM); (L) Maximum Entropy Model (MaxEnt).

Page S9

Table S2. Comparison of evaluation criteria for 12 species distribution models. Bold font represented the final prediction model.

Page S10

Figure S3. Results of three evaluation metrics of 56 MaxEnt models run using different combinations of regularization multiplier and feature combination: (a) Minimum information criterion AICc values. (b) Difference between training AUC and test AUC ($AUC_{train} - AUC_{test}$) values. (c) 10% test omissionrate values. The default parameters and the optimal parameters were indicated by black and red arrows respectively. Feature classes: L-linear; H-hinge; Q-quadratic; P-product; and T-threshold.

Page S11

Table S3. Evaluation metrics of the MaxEnt model generated by ENMeval.
FC: Feature Combination；RM: Regularization Multiplier；AICc: The akaike information criterion corrected: AUC: The area under the receiver operating characteristic curve; LQHPT: Linear features(L) + Quadratic features(Q) + Hinge features(H) + Product features(P) + Threshold features(T); Delta.AICc: The minimum information criterion AICc value; $Avg.AUC_{DIFF:}$ Difference between the AUC values; Or.10pct: 10% test omissionrate; SD: standard deviation.

Page S12

Figure S4. The relative importance of different predictor variables based on the results of the jackknife test in MaxEnt. The graphs show the contributions of the variables: (A) Regularized training gain and (B) Test gain (C) AUC.

Page S13

Figure S5. Relationships between dominant environmental variables and suitability probability of tribe Erythroneurini.
Notes: Response curves illustrated how the predicted probability of presence changes when the model was created using only that variable. The curves show the mean response of the 10 replicate Maxent runs (red) and and the mean +/- one standard deviation (blue). (A) BIO6 (Min temperature of coldest month) and (B) BIO4 (Temperature seasonality) and (C) BIO12 (Annual precipitation) and (D) BIO2 (Mean diurnal air temperature area). RTG：Regularized Training Gain. PC:Percent Contribution.

Page S14

Table S4. Centroid distributional shifts under different climate scenario/periods for tribe Erythroneurini.

Table S1. The distribution points of tribe Erythroneurini for modeling. Note: the distribution data of the Erythroneurini screened out through steps such as collection, correction, and sparsification.

genera	*Species*	*Longitude/°*	*Latitude/°*
Andrabia Ahmed, 1970	*Alnetoidia alneti (Dahlbom,1850)*	*121.2959*	*24.2723*
	Alnetoidia cedrelae Chou et Ma,1981	*108.0741*	*34.2816*
	Alnetoidia dentata Cao, Yang & Zhang, 2016	*97.7995*	*24.0683*
	Alnetoidia dujuanensis Song et Li, 2010	*105.6975*	*28.5905*
	Alnetoidia sikkimensis Dworakowska, 1994	*97.7069*	*24.7544*
	Alnetoidia sudzuchenica (Anufriev, 1971)	*116.7296*	*40.1329*
	Alnetoidia triseta Dworakowska, 1994	*116.9155*	*40.2123*
	Alnetoidia gracilis Cao, Yang et Zhang, 2016	*103.5601*	*30.9034*
Anufrievia Dworakowska, 1970	*Anufrievia adaucta Cao & Zhang, 2018*	*117.6991*	*27.7773*
	Anufrievia bauhinicola Dworakowska & Viraktamath, 1978	*99.1023*	*25.127*
	Anufrievia drepanoides Lin & Zhang, 2021	*114.3672*	*30.5519*
	Anufrievia liubana Yang & Zhang, 2018	*108.9365*	*30.291*
	Anufrievia maculosa Dworakowska,1977	*107.2001*	*35.6815*
	Anufrievia qinlingensis Yang & Zhang, 2018	*108.3268*	*34.0508*
	Anufrievia subdentata Yang & Zhang, 2018	*112.991*	*25.7401*
	Anufrievia triprocessa Yang & Zhang, 2018	*103.6268*	*28.236*
Anuihuajianga Zhang & Song, 2022	*Anuihuajianga pyramidalis Zhang & Song, 2022*	*106.0973*	*25.1743*
Arboridia Zakhvatkin, 1946	*Arboridia apicalis (Nawa, 1913)*	*87.6007*	*43.7732*
		89.0716	*43.1468*
	Arboridia agrillacea (Anufriev, 1969)	*97.0063*	*33.0063*
		104.2151	*31.741*
		106.5312	*32.6337*
		109.0236	*33.9685*
		112.4527	*35.2647*
	Arboridia apicalis (Nawa, 1913)	*119.5289*	*39.884*
		123.435	*41.8426*
	Arboridia cihuashana (Song et Li, 2013)	*109.9955*	*34.5279*
	Arboridia maculifrons (Vilbaste, 1968)	*115.7108*	*37.5304*
		116.4166	*39.8781*
	Arboridia suputinkaensis (Vilbaste, 1968)	*118.9069*	*30.1081*
	Arboridia suzukii Dworakowska,1916	*108.2356*	*33.8926*
	Arboridia luojiashangensis Zhang, Jiang & Song, 2022	*108.6436*	*27.912*
	Arboridia ochracea Song & Li, 2015	*111.8387*	*33.6681*
	Arboridia surstyli Cai & Xu, 2006	*119.1179*	*29.8481*
	Arboridia tridentata (Song et Li, 2013)	*99.4195*	*26.4529*
Balanda Dworakowska, 1979	*Balanda kara Dworakowska, 1979*	*100.9885*	*22.7548*
Chagria Dworakowska, 1994	*Chagria camptoprocessa Cao, Huang et Zhang, 2011*	*100.8939*	*22.0887*

Coganoa Dworakowska, 1976	*Coganoa arcuata Dworakowska, 1976*	*109.5394*	*18.2661*
Coloana Dworakowska, 1971	*Coloana bifurcata Sohi & Mann, 1992*	*109.5028*	*18.9347*
	Coloana hainanensis Yang & Zhang, 2014	*109.6626*	*18.8833*
		116.6067	*40.6501*
Davmata Dworakowska, 1979	*Davmata falcata Cao, Huang et Zhang, 2011*	*100.8537*	*22.1701*
Dentaneura Song, Li & Dai, 2016	*Dentaneura henanensis Song, Li & Dai, 2016*	*106.6722*	*26.4329*
Diomma Motschulsky, 1863	*Diomma ulae (Dworakowska, 1972)*	*106.6383*	*26.6432*
	Diomma knighti Dworakowska, 1981	*120.8277*	*24.1198*
	Diomma taiwana Shiraki, 1912	*104.4757*	*25.6981*
		109.1706	*19.0638*
	Diomma katoi Dworakowska, 1981	*108.0691*	*25.3896*
	Diomma pincersa Song, Li & Xiong, 2011	*105.9149*	*27.2264*
		110.0953	*29.7817*
	Diomma pulchrum (Matsumura, 1916)	*106.6825*	*26.3761*
		107.9018	*28.5554*
Dorycnia Dworakowska, 1972	*Dorycnia vietnamica Dworakowska, 1972*	*97.6152*	*24.6087*
Duanjina Kuoh, 1981	*Duanjina liangdiana Kuoh, 1981*	*98.5935*	*29.68*
Elbelus Mahmood, 1967	*Elbelus tripunctatus Mahmood,1967*	*97.895*	*24.8369*
		106.4138	*23.7239*
		107.3169	*25.8297*
		108.3207	*23.4984*
		108.3798	*22.7874*
		109.5352	*19.077*
		110.1904	*24.1305*
		114.2845	*26.3155*
		120.1489	*30.264*
		129.6329	*44.5515*
	Elbelus wierzbowskae Dworakowska, 1972	*104.9519*	*25.1277*
Empoascanara Distant, 1918	*Empoascanara alami Ahmed, 1970*	*108.7881*	*19.1091*
	Empoascanara arcuata Song & Li, 2014	*106.3974*	*29.837*
	Empoascanara dwalata Dworakowska ,1977	*114.413*	*30.5311*
	Empoascanara hongkongica Dworakowska ,1971	*109.4955*	*19.5064*
	Empoascanara kotoshonis Matsumura, 1940	*120.7027*	*24.0583*
	Empoascanara limbata Matsumura, 1907	*108.2538*	*27.9375*
	Empoascanara maculifrons Motschulsky, 1863	*110.5629*	*19.9535*
		119.6623	*27.711*
		121.4736	*31.2314*
	Empoascanara penta Dworakowska, 1992	*102.935*	*24.5197*
		106.6917	*29.605*
		112.2827	*35.1877*

	Empoascanara sipra Dworakowska, 1980	106.2547	25.6864
		107.1484	30.8882
		114.0764	31.8121
	Empoascanara apara Dworakowska, 1979	109.7702	19.1729
	Empoascanara circumscripta (Matsumura, 1910)	91.0993	29.6726
	Empoascanara nigrobimaculata (Motschulsky, 1863)	107.1755	28.205
Erythroneura Fitch, 1851	*Erythroneura elegantula* Osborn, 1928	103.6755	22.9867
Etmaria Cao & Dmitriev, 2020	*Etmaria sinuata* (Chiang & Knight, 1990)	120.6908	23.9012
Fractata Song & Li, 2011	*Fractata sinuata* Song et Li, 2011	101.543	21.2814
Frutioidia Zakhvatkin, 1946	*Frutioidia nefara* Dworakowska, 1979	108.4254	28.6252
Gambialoa Dworakowska, 1972	*Gambialoa asiatica* Dworakowska, 1979	101.5463	21.7238
Gladkara Dworakowska, 1995	*Gladkara albida* Dworakowska, 1995	101.5716	21.4892
Gredzinskiya Dworakowska，1972	*Gredzinskiya bipunctata* Chiang et Knight, 1990	120.9285	24.7711
Hepneriana Dworakowska, 1972	*Hepneriana applanata* Yang, Cao & Zhang, 2016	102.7736	25.1395
Helionidia Zakhvatkin, 1946	*Helionidia ochrata* Dworakowska, 1970	120.2996	22.726
Hamata Cao, Dmitriev, Dietrich & Zhang, 2019	*Hamata coralliformis* Cao, Dmitriev, Dietrich & Zhang, 2019	100.7996	22.0118
Irenaneura Cao, Huang & Zhang, 2012	*Irenaneura dworakowski* Cao, Huang et Zhang, 2012	100.8914	22.2535
Kabakra Dworakowska, 1979	*Kabakra acutata* Cao, Huang et Zhang, 2012	100.8832	22.0269
Kapsa Dworakowska, 1972	*Kapsa dolka* Dworakowska, 1979	98.8034	25.2786
		102.1766	28.5501
		115.9699	39.6956
		116.8603	25.2705
	Kapsa huajiangensis Yang, Luo & Song, 2022	105.8116	24.9837
	Kapsa aculeiformis Cao et Zhang, 2013	94.0878	29.8128
	Kapsa brevis Cao et Zhang, 2013	94.916	30.1802
	Kapsa explanata Cao et Zhang, 2013	94.2131	29.216
	Kapsa megaprocessa Cao et Zhang, 2013	94.4032	29.6197

Kaukania Dworakowska, 1972	*Kaukania anser Dworakowska, 1972*	*100.9222*	*22.286*
Kanguza Dworakowska, 1972	*Kanguza peckera (Song & Li, 2014)*	*118.8662*	*32.0708*
Kusala Dworakowska, 1981	*Kusala datianensis Song et Li, 2011*	*109.7173*	*18.6881*
Laciniata Song & Li, 2013	*Laciniata lijianga Song et Li, 2013*	*100.234*	*26.8889*
Lectotypella Dworakowska, 1972	*Lectotypella albisoma (Matsumura, 1932)*	*120.3105*	*22.6984*
		120.5782	*24.1401*
Matsumurina Dworakowska, 1972	*Matsumurina kagina (Matsumura, 1932)*	*120.3338*	*22.8154*
	Matsumurina jianfenga Song et Li, 2011	*121.0433*	*24.2875*
	Matsumurina macra Kuoh, 1982	*114.2404*	*26.4084*
		115.8166	*28.6372*
		118.6537	*36.2394*
Mitjaevia Dworakowska, 1970	*Mitjaevia acrodonta Lin & Zhang, 2022*	*97.9356*	*24.7119*
	Mitjaevia acrodonta Lin & Zhang, 2022	*110.355*	*21.2709*
	Mitjaevia diana (Distant, 1918)	*98.7292*	*24.9772*
	Mitjaevia nanaoensis Chiang et Knight, 1990	*108.0777*	*26.3546*
	Mitjaevia salaxia Luo, Wang & Song, 2022	*107.8055*	*25.2583*
	Mitjaevia tappana Chiang et Knight, 1990	*95.0697*	*30.0949*
Motaga Dworakowska, 1979	*Motaga rokfa Dworakowska, 1979*	*100.2857*	*21.9045*
	Motaga rokfaoides Lin & Zhang, 2023	*101.5876*	*21.5962*
Niedoida Dworakowska, 1994	*Niedoida atrifrons Distant, 1918*	*118.8217*	*32.063*
Ossuaria Dworakowska, 1979	*Ossuaria sichuanensis Zhang et Yang, 2011*	*103.4075*	*29.5756*
	Ossuaria yunnanensis Zhang et Yang, 2011	*101.2739*	*21.9172*
Plumosa Sohi, 1977	*Plumosa nigrimaculata Song et Li, 2008*	*109.8304*	*18.7285*
Pseudothaia Kuoh, 1982	*Pseudothaia striata Kuoh, 1982*	*110.1977*	*18.7355*
Qadria Mahmood, 1967	*Qadria daliensis Song et Li, 2014*	*98.7148*	*26.1087*
		100.1989	*25.7039*
	Qadria dongfangensis Song et Li, 2014	*109.5588*	*18.8312*
	Qadria pakistanica Ahmed, 1969	*120.9111*	*23.8649*
Raabeina Dworakowska, 1972	*Raabeina acutata Zhang et Cao, 2011*	*95.7626*	*29.8305*
	Raabeina curtihamata Zhang et Cao, 2011	*102.6252*	*24.9735*
	Raabeina fuscofasciata Dworakowska, 1972	*103.2327*	*22.8125*
	Raabeina hsui Chiang et Knight, 1990	*113.9054*	*22.7572*
	Rufitidia forficata Song et Li, 2009	*108.0349*	*25.2346*

Rufitidia Dworakowska, 1994		*112.5137*	*27.9107*
Sanatana Dworakowska, 1984	*Sanatana malaica Dworakowska，1984*	*107.9778*	*26.5572*
Saccata Cao & Zhang, 2013	*Saccata insolita Cao et Zhang,2013*	*100.6821*	*21.5781*
Salka Dworakowska, 1972	*Salka abbotta Chiang et Knight, 1990*	*106.0549*	*32.7978*
		108.3448	*26.3374*
	Salka addonica Chiang et Knight, 1990	*121.564*	*24.8054*
	Salka arenaria Sohi et Mann, 1994	*121.5862*	*25.1466*
	Salka cambera Song et Li, 2015	*111.8963*	*33.8403*
	Salka cerviprocessa Song et Li, 2012	*98.4807*	*25.029*
	Salka congjianga Song et Li, 2012	*108.5219*	*25.9319*
	Salka guilinensis Song et Li, 2012	*109.9352*	*25.5983*
	Salka kerzhneri Dworakowska,2006	*97.8065*	*24.8031*
	Salka musica Sohi et Mann, 1994	*120.8262*	*23.575*
Seriana Dworakowska, 1971	*Seriana indefinita Dworakowska， 1971*	*106.0374*	*32.9667*
		107.1279	*29.0489*
		114.1436	*22.3994*
	Seriana ochrata Dworakowska，1971	*101.38*	*21.7093*
Singapora Mahmood, 1967	*Singapora arifi Ghauri, 1985*	*97.9243*	*24.6082*
	Singapora candela Yang et Zhang, 2014	*107.1918*	*21.9963*
	Singapora fopingensis Chou et Ma, 1981	*113.2676*	*23.1239*
		119.2962	*26.1011*
	Singapora shiqianensis Lin & Zhang, 2020	*106.8052*	*26.5378*
	Singapora karnatakana Viraktamath et Dworakowska, 1979	*98.7529*	*24.8684*
	Singapora shinshana Matsumura, 1932	*104.1056*	*30.6563*
		113.0671	*36.2025*
	Singapora yingjiangica Cao et Zhang, 2014	*97.7531*	*24.4937*
Tamaricella Zakhvatkin, 1946	*Tamaricella fuscula Cai, 1999*	*89.2432*	*42.9873*
		109.8885	*40.6409*
Tautoneura Anufriev, 1969	*Tautoneura ahmedi Dworakowska, 1977*	*100.2431*	*25.6027*
	Tautoneura ahmedi Dworakowska, 1977	*108.2428*	*28.7935*
	Tautoneura arachisi Matsumura, 1916	*106.7669*	*25.4334*
		117.9597	*27.6564*
	Tautoneura choui Ma, 1983	*107.018*	*30.8265*
	Tautoneura elscinta Chiang et Knight, 1990	*104.9745*	*27.2455*
	Tautoneura formosa Dworakowska, 1970	*120.1213*	*30.2227*
	Tautoneura fusca Dworakowska, 1970	*113.3459*	*22.0561*
	Tautoneura mori Matsumura, 1906	*104.761*	*32.5811*
		116.4464	*36.2933*
		120.2141	*30.2454*
	Tautoneura puerensis Song et Li, 2012	*102.1356*	*29.6461*
	Tautoneura sanguinalis Distant, 1918	*117.2293*	*36.5341*
	Tautoneura sinica Dworakowska, 1970	*113.5536*	*22.3149*
	Tautoneura takaonella Matsumura, 1932	*120.769*	*23.1575*

	Tautoneura tengchongna Song & Li, 2014	*98.8935*	*24.91*
	Tautoneura tripunctula Melicher, 1903	*106.7508*	*26.5465*
	Tautoneura unicolor Dworakowska, 1979	*108.1806*	*28.652*
	Tautoneura yunnanensis Song, Xiong et Li, 2011	*99.9362*	*22.5647*
Thaia Ghauri, 1962	*Thaia lincanga Song & Li, 2014*	*99.7918*	*24.7673*
	Thaia bimaculata (Kuoh, 1982)	*115.9731*	*29.522*
	Thaia infumata Kuoh, 1982	*117.2733*	*27.0845*
	Thaia maxima Dworakowska, 1976	*120.4691*	*23.3189*
	Thaia nigra Dworakowska, 1970	*114.0165*	*22.3979*
	Thaia oryzivora Ghauri, 1962	*97.6617*	*24.6016*
		112.5391	*23.1677*
	Thaia subrufa (Motschulsky, 1863)	*106.1972*	*28.3329*
		114.5285	*24.6241*
Thailus Mahmood, 1967	*Thailus versicolor Cao et Zhang, 2013*	*99.8067*	*22.7138*
	Thailus versicolor Cao et Zhang, 2013	*107.8167*	*26.0775*
Thapaia Dmitriev & Dietrich, 2006	*Thapaia plumula Song et Li, 2009*	*103.775*	*29.5435*
	Thapaia tibetensis Cao, Dmitriev, Dietrich & Zhang, 2019	*94.6533*	*29.6396*
Variolosa Cao & Zhang, 2013	*Variolosa meni Cao et Zhang, 2013*	*109.8664*	*19.0949*
Watara Dworakowska, 1977	*Watara cordata Zhang et Yang, 2011*	*100.2811*	*23.933*
	Watara sudra (Distant, 1980)	*104.2064*	*26.8773*
Yakuza Dworakowska, 2002	*Yakuza centralis Dworakowska, 2002*	*120.687*	*22.9684*
	Yakuza sumatrana Dworakowska, 2002	*103.6061*	*31.0168*
Ziczacella Anufriev, 1970	*Ziczacella dworakowskae (Anufriev, 1970)*	*103.1434*	*31.1403*
	Ziczacella heptapotamica (Kusnezov, 1928)	*103.0415*	*30.01*
		112.739	*27.2565*
	Ziczacella hirayamella (Matsumura, 1931)	*116.1521*	*39.7946*
		116.4404	*39.9162*
	Ziczacella lyrifora (Dlabola, 1968)	*112.0774*	*38.8312*
	Ziczacella steggerdai (Ross,1965)	*102.9749*	*29.6736*
		104.2537	*26.8575*
		108.8923	*34.0392*
Zygina Fieber, 1866	*Zygina biprocessa Song & Li, 2014*	*117.2263*	*37.4221*
	Zygina flammena Song & Li, 2014	*107.1801*	*28.9393*
	Zygina bipunctula (Melichar, 1903)	*107.4545*	*28.5535*
		107.6075	*29.1512*
	Zygina discolor Horvath, 1897	*108.905*	*25.7539*
	Zygina hazatrnsis Ahmed, 1970	*108.2784*	*26.4394*
Zyginopsis Ramakrishnan & Menon, 1973	*Zyginopsis verticalis (Ahmed, 1970)*	*108.8623*	*18.7386*

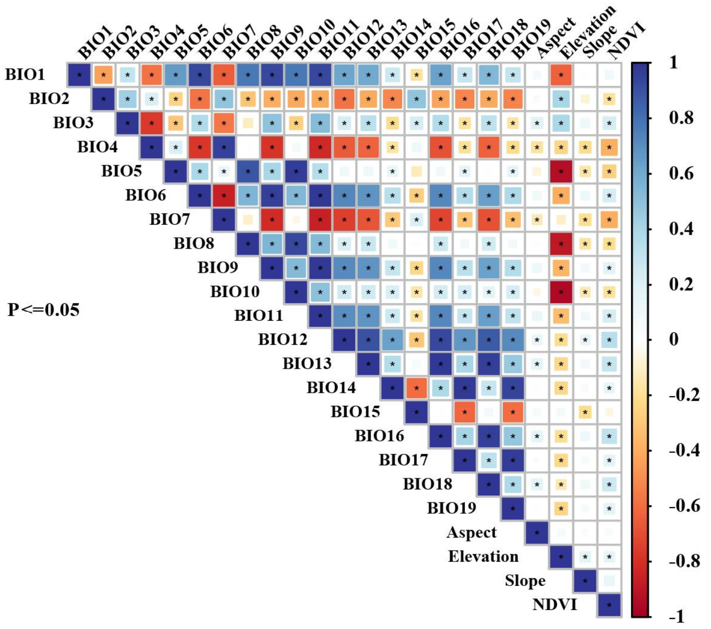

(a)

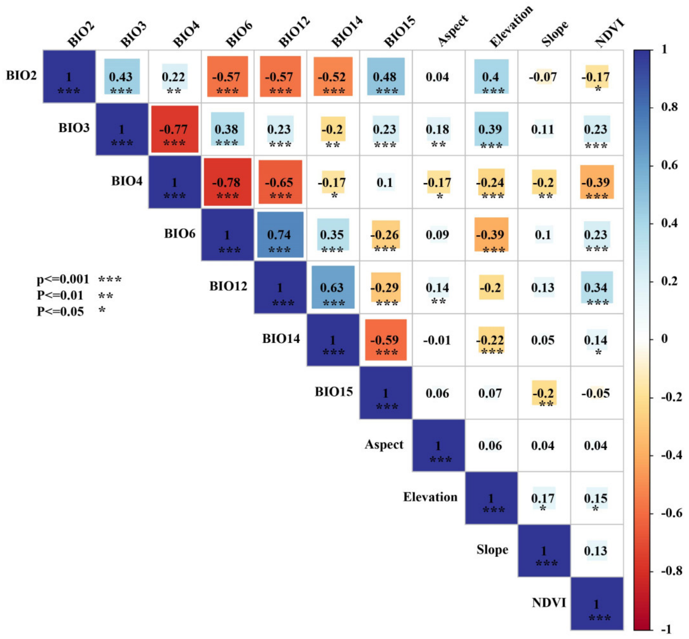

(b)

Figure S1. (a) Pearson correlation analysis among the 23 environmental variables. **(b)** Multicollinearity test of Pearson correlation analysis between environmental variables retained for MaxEnt modeling. Note: Environmental variables with Pearson correlation coefficient less than |0.8| were retained for the next step of operation.

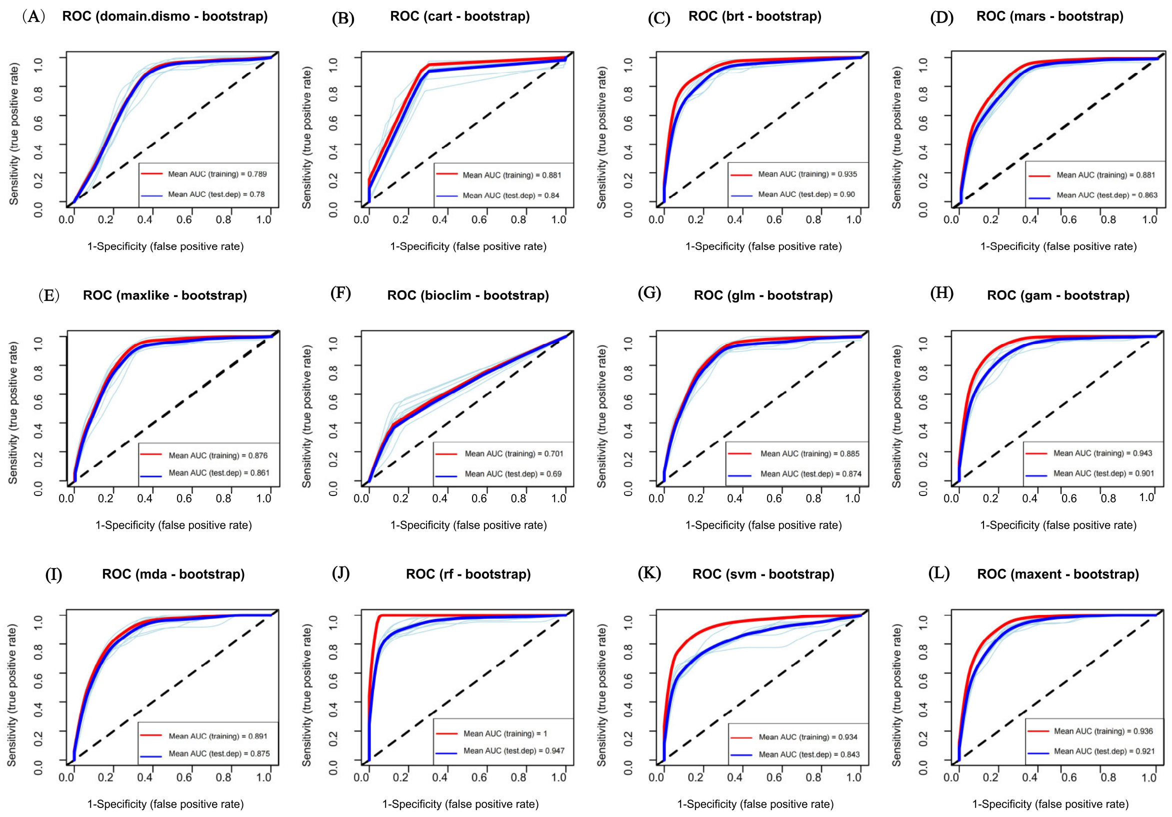

Figure S2. The receiver operating characteristic (ROC) curves of the 12 species distribution models: (A) Domain; (B) Classification and Regression Trees (CART); (C) Boosted Regression Trees; (D) Multivariate Adaptive Regression Spline (MARS); (E) Maxlike; (F) Bioclim; (G) Generalized Linear Model (GLM); (H) Generalized Additive Model (GAM); (I) Mixture Discriminant Analysis (MAD); (J) Random Forests (RF); (K) Support Vector Machine (SVM); (L) Maximum Entropy Model (MaxEnt).

Table S2. Comparison of evaluation criteria for 12 species distribution models. Bold font represented the final prediction model.

**Methods**	**AUC_{test} (±SD)**	**TSS (±SD)**	**$Avg.AUC_{DIFF}$**
Domain.dismo	0.78 ± 0.021	0.54 ± 0.046	0.009
CART	0.84 ± 0.033	0.61 ± 0.050	0.041
BRT	0.90 ± 0.013	0.67 ± 0.030	0.035
MARS	0.86 ±0.019	0.62 ± 0.028	0.018
MaxLike	0.86 ± 0.016	0.63 ± 0.026	0.015
BIOCLIM	0.69 ± 0.034	0.39 ± 0.071	0.011
GLM	0.87 ± 0.017	0.63 ± 0.024	0.018
GAM	0.90 ± 0.015	0.67 ± 0.027	0.042
MDA	0.88 ± 0.014	0.63 ± 0.039	0.016
RF	0.95 ± 0.007	0.78 ± 0.022	0.053
SVM	0.84 ± 0.040	0.58 ± 0.068	0.091
**MaxEnt**	**0.92 ± 0.012**	**0.69 ± 0.039**	**0.015**

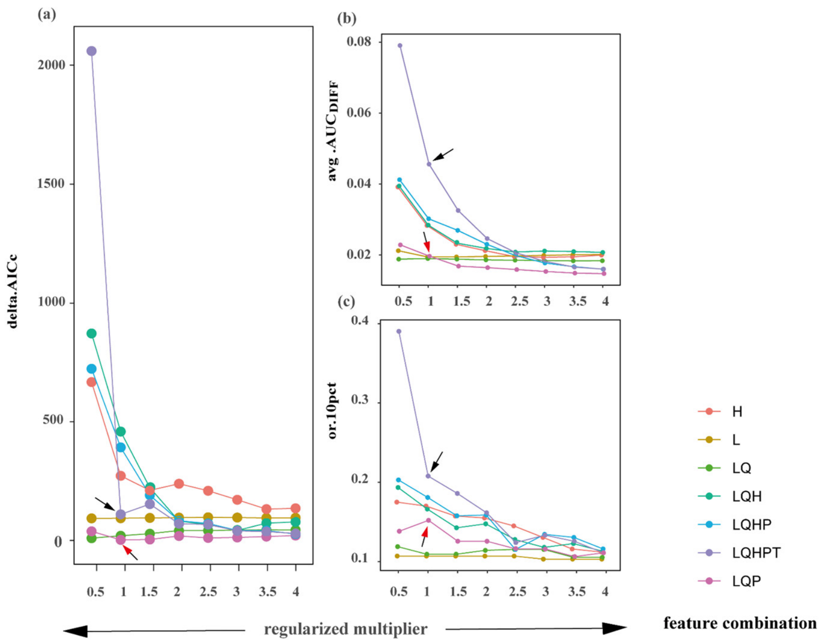

Figure S3. Results of three evaluation metrics of 56 MaxEnt models run using different combinations of regularization multiplier and feature combination: (a) Minimum information criterion AICc values. (b) Difference between training AUC and test AUC ($AUC_{train} - AUC_{test}$) values. (c) 10% test omissionrate values. The default parameters and the optimal parameters were indicated by black and red arrows respectively. Feature classes: L-linear; H-hinge; Q-quadratic; P-product; and T-threshold.

Table S3. Evaluation metrics of the MaxEnt model generated by ENMeval.

Type	Feature Combination	Regularization Multiplier	Delta.AICc	Avg.AUC_{DIFF}	Or.10pct	AUCtrain (±SD)
Default	LQPHT	1	106.07	0.0456	0.2079	0.949 ±0.005
Optimized	LQP	1	0	0.0196	0.1519	0.911 ±0.005

FC: Feature Combination；RM: Regularization Multiplier；AICc: The Akaike information criterion corrected: AUC: The area under the receiver operating characteristic curve; LQHPT: Linear features(L) + Quadratic features(Q) + Hinge features(H) + Product features(P) + Threshold features(T); Delta.AICc: The minimum information criterion AICc value; Avg.$AUC_{DIFF:}$ Difference between the AUC values; Or.10pct: 10% test omissionrate; SD: standard deviation.

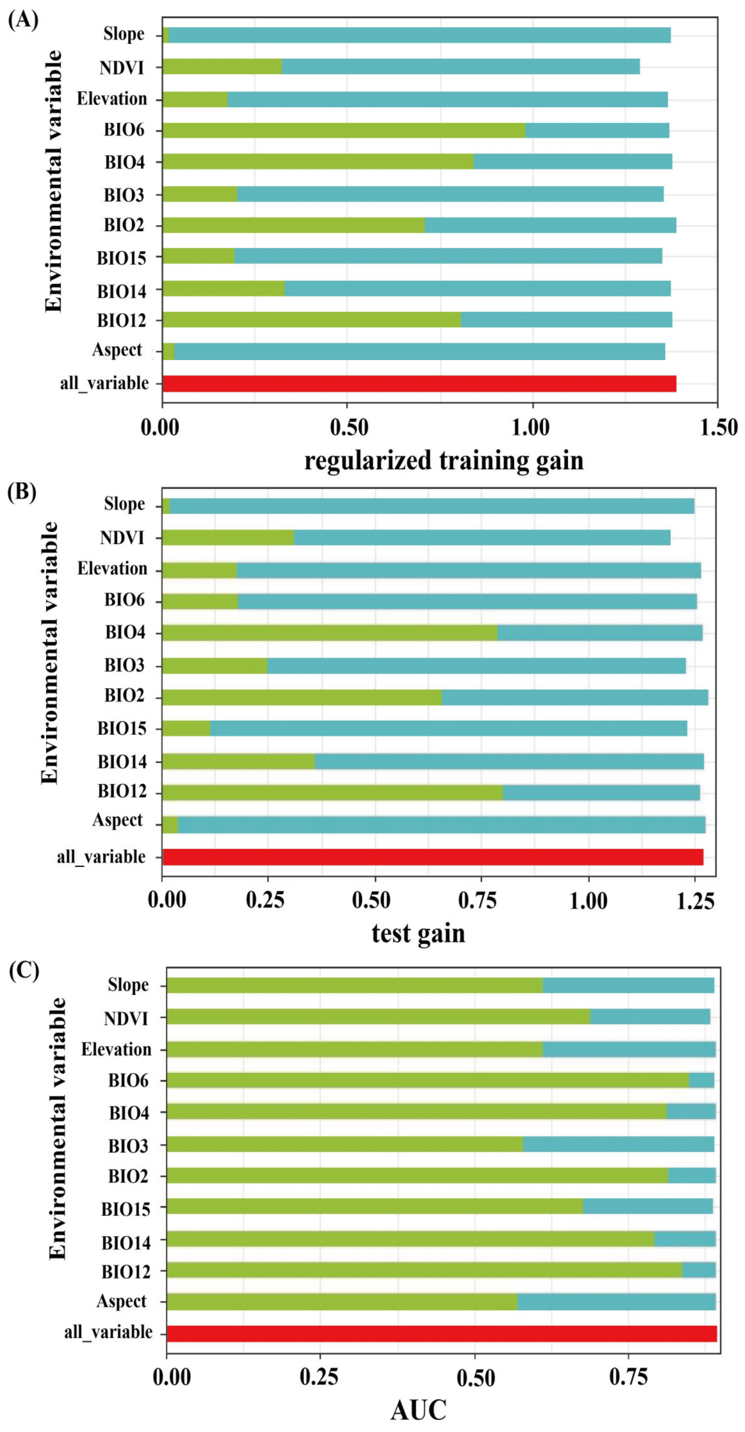

Figure S4. The relative importance of different predictor variables based on the results of the jackknife test in MaxEnt. The graphs show the contributions of the variables: (A) Regularized training gain and (B) Test gain (C) AUC.

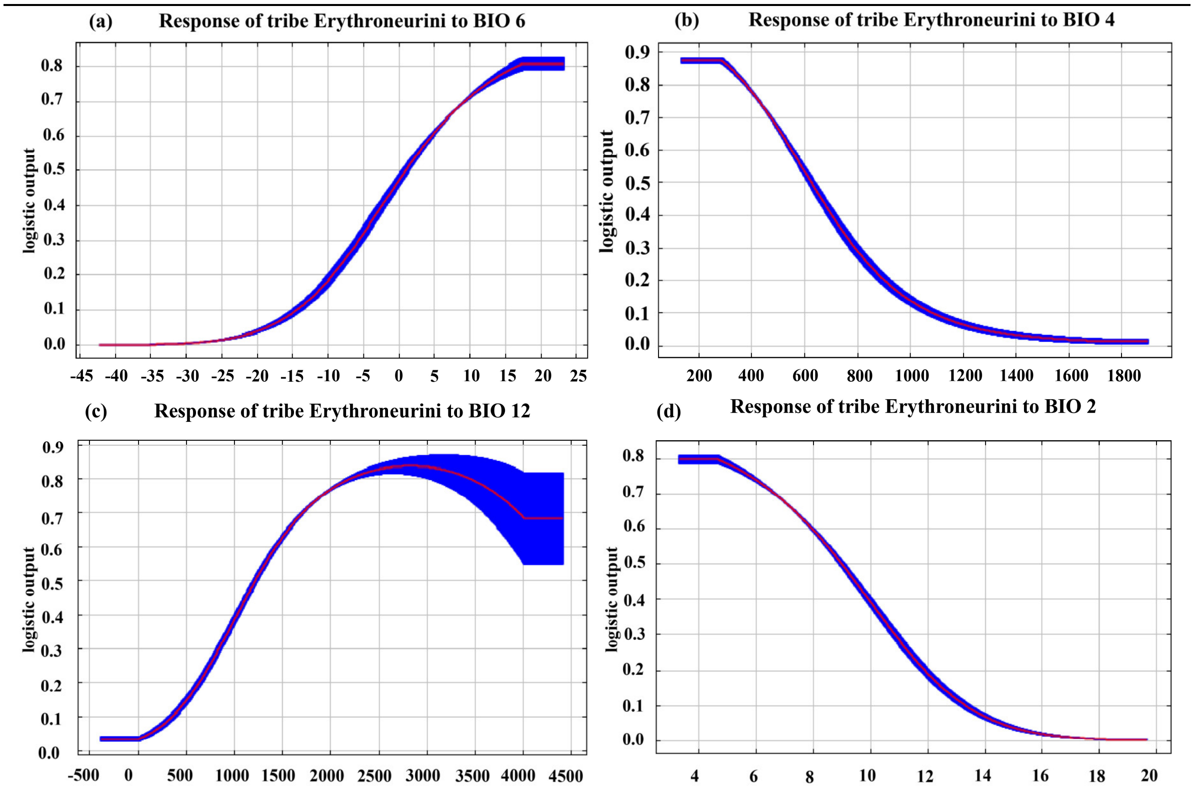

Figure S5. Relationships between top bioclimatic variables and probability of tribe Erythroneurini presence. Notes: Response curves illustrated how the predicted probability of presence changes when the model was created using only that variable. The curves show the mean response of the 10 replicate Maxent runs (red) and and the mean +/- one standard deviation (blue). (A) BIO6 (Min temperature of coldest month) and (B) BIO4 (Temperature seasonality) and (C) BIO12 (Annual precipitation) and (D) BIO2 (Mean diurnal air temperature area). RTG：Regularized Training Gain. PC: Percent Contribution.

Table S4. Centroid distributional shifts under different climate scenarios/periods for tribe Erythroneurini.

Period	Historical climate scenarios			Future climate scenarios					
	LGM	MH	Current	SSP126 2050s	SSP126 2070s	SSP126 2090s	SSP585 2050s	SSP585 2070s	SSP585 2090s
Longitude/**(°)**	114.00	113.44	113.93	114.30	114.17	113.62	113.71	113.08	114.33
Latitude/**(°)**	31.61	31.44	32.57	32.10	32.37	31.94	31.79	32.92	31.99
Elevation/**m**	95	104	152	94	88	155	156	138	93
Distance/**km**	-	56.99	134.04	62.61	31.90	70.22	89.18	138.56	156.38
